# Machine learning-based detection of immune-mediated diseases from genome-wide cell-free DNA sequencing datasets

**DOI:** 10.1038/s41525-022-00325-w

**Published:** 2022-09-14

**Authors:** Huiwen Che, Tatjana Jatsenko, Lore Lannoo, Kate Stanley, Luc Dehaspe, Leen Vancoillie, Nathalie Brison, Ilse Parijs, Kris Van Den Bogaert, Koenraad Devriendt, Sabien Severi, Ellen De Langhe, Severine Vermeire, Bram Verstockt, Kristel Van Calsteren, Joris Robert Vermeesch

**Affiliations:** 1 Department of Human Genetics, Laboratory for Cytogenetics and Genome Research, KU Leuven, Leuven, Belgium; 2 Department of Gynecology and Obstetrics, University Hospitals Leuven, Leuven, Belgium; 3 Centre for Human Genetics, University Hospitals Leuven, Leuven, Belgium; 4 Department of Rheumatology, University Hospitals Leuven, Leuven, Belgium; 5 Department of Development and Regeneration, Laboratory of Tissue Homeostasis and Disease, KU Leuven, Leuven, Belgium; 6 Department of Gastroenterology and Hepatology, University Hospitals Leuven, Leuven, Belgium; 7 Department of Chronic Diseases and Metabolism, TARGID-IBD, KU Leuven, Leuven, Belgium

**Keywords:** Autoimmune diseases, Medical genomics, Data mining, Personalized medicine

## Abstract

The early detection of tissue and organ damage associated with autoimmune diseases (AID) has been identified as key to improve long-term survival, but non-invasive biomarkers are lacking. Elevated cell-free DNA (cfDNA) levels have been observed in AID and inflammatory bowel disease (IBD), prompting interest to use cfDNA as a potential non-invasive diagnostic and prognostic biomarker. Despite these known disease-related changes in concentration, it remains impossible to identify AID and IBD patients through cfDNA analysis alone. By using unsupervised clustering on large sets of shallow whole-genome sequencing (sWGS) cfDNA data, we uncover AID- and IBD-specific genome-wide patterns in plasma cfDNA in both the obstetric and general AID and IBD populations. We demonstrate that pregnant women with AID and IBD have higher odds of receiving inconclusive non-invasive prenatal screening (NIPS) results. Supervised learning of the genome-wide patterns allows AID prediction with 50% sensitivity at 95% specificity. Importantly, the method has the potential to identify pregnant women with AID during routine NIPS. Since AID pregnancies have an increased risk of severe complications, early recognition or detection of new-onset AID can redirect pregnancy management and limit potential adverse events. This method opens up new avenues for screening, diagnosis and monitoring of AID and IBD.

## Introduction

Increased levels of cell-free DNA (cfDNA) have been observed in individuals with systemic lupus erythematosus (SLE), a common and prototypical autoimmune disease (AID), as early as the 1960s^1^. The elevation of cfDNA has been found in other AID, such as rheumatoid arthritis (RA)^2^ and inflammatory bowel disease (IBD), including Crohn’s disease (CD) and ulcerative colitis (UC)^3,4^. Despite the existence of different predictive and prognostic biomarkers for AID and IBD, more robust non-invasive markers to deliver precision medicine are needed^5,6^. Given the observed elevation, cfDNA has become a promising biomarker for prediction, monitoring, and treatment response stratification for AID and IBD^7–11^. Although assessment of overall cfDNA concentrations in these diseases allows for the inference of AID and IBD activity, it lacks specificity and provides no information about qualitative disease-associated cfDNA changes, which might aid in disease diagnosis or prognosis.

CfDNA analysis has been clinically implemented for prenatal^12^ and cancer^13^ management. Non-invasive prenatal screening (NIPS) for aneuploidies detection has become a cornerstone of prenatal care over the past decade^14^. Placenta-derived cfDNA is present in maternal plasma and serum^15^, and different approaches have been developed to detect fetal chromosomal anomalies^12,16^. Despite the high accuracy of NIPS, approximately 0.2–5.4% of NIPS remain inconclusive, even after repeated testing^17–21^. Multiple studies have reported that the most common reason for inconclusive NIPS results is a low fetal fraction (FF), accounting for up to 50% of test failures^22–24^. A low FF can be due to either a low amount of placental DNA (e.g. early gestational age) or to an increased amount of maternal DNA. A number of maternal conditions can increase the maternal cfDNA fraction^22,25^. Recently, AID has been identified as a contributor to reduced FF^22,26–28^. As a consequence, pregnant AID patients have significantly higher odds of receiving an inconclusive NIPS result when compared to the general obstetric population^24,26^.

The growing volume of shallow whole-genome cfDNA sequencing (sWGS) data permits the exploration of disease-associated cfDNA signatures using machine learning approaches. By applying GIP*Xplore*^29^, our recently developed algorithm for sWGS data mining using unsupervised clustering and supervised machine learning, we have already demonstrated the presence of cancer-specific patterns in plasma cfDNA profiles from cancer patients. Therefore, we hypothesized that autoimmune-related disease patterns might equally be elucidated via this approach. By interrogating over 80,000 cfDNA profiles from pregnant women and an independent cohort of non-pregnant individuals, we revealed AID- and IBD-associated genome-wide cfDNA signatures that allow the detection of the disease both during pregnancy and in non-pregnant patients. This study demonstrates that sWGS of cfDNA can identify women with immune-mediated diseases early during pregnancy, having a potential utility to detect, diagnose and monitor individuals at high risk. Our approach can be easily integrated with routine NIPS workflows and pending success in large-scale prospective trials can be broadly implemented for better prenatal management.

## Results

### AID and IBD are overrepresented in inconclusive NIPS and are characterized by recurrent cfDNA profile patterns

We analyzed 81,611 cfDNA samples from pregnant women and identified a total 185 inconclusive cases (0.23%) using an unbiased NIPS analysis pipeline coined GIPSeq^16^. The inconclusive result (Cohort I) was attributed to either a high variance in genome-wide cfDNA read counts (high quality score (QS); *n* = 143; 77%) or a low fetal fraction (low FF; *n* = 42; 23%). Since all the inconclusive cases had a second sampling, and for some, an additional third technical replicate, a total of 406 cfDNA profiles were obtained from the 185 pregnant women.

**Fig. 1:**
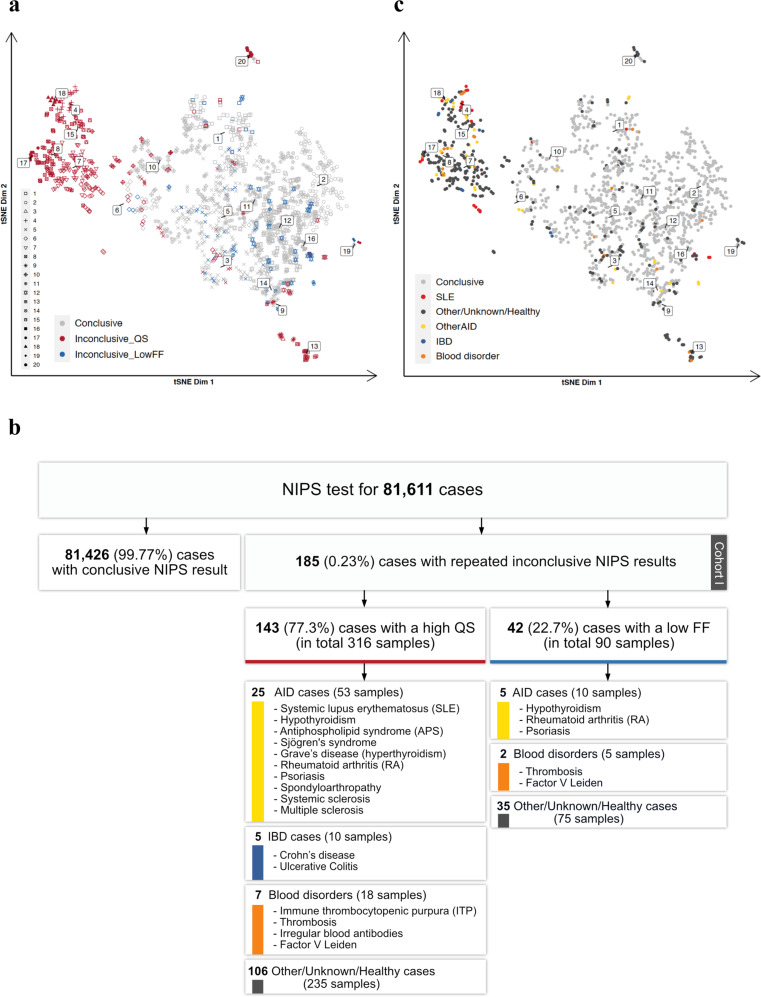
Clustering of repeated inconclusive NIPS. **a** tSNE representation of inconclusive samples (*n* = 406) and conclusive NIPS controls (*n* = 1024). Each point represents one sample. Colours red and blue indicate inconclusive samples due to deviating QS and low FF respectively. Point shape represents the clusters being identified using Walktrap community detection. **b** overview of the study cohort and cases with immune-mediated diseases. **c** tSNE representation with clinical information annotation.

Using GIP*Xplore*, we performed explorative analysis on the set of inconclusive NIPS profiles and randomly selected conclusive NIPS control samples. Two to three conclusive control samples were matched to the sample processing time, sequencing batch and library preparation kit of inconclusive cases, accumulating to 1024 samples. The unsupervised clustering revealed a separation between a subset of the inconclusive profiles with deviating QS and conclusive NIPS profiles (Fig. 1a). Most of the inconclusive profiles with deviating QS fell into two large groups, where clusters 4, 15, and 18 formed one group and clusters 7, 8 and 17 composed another one with similar genome-wide profiles (Supplementary Fig. 1). A few scattered cases could be found in clusters 13, 19 and 20. The inconclusive profiles with low FF, in general, overlapped with conclusive profiles. Repeated samples, both technical and biological replicates, from the same inconclusive case, showed highly concordant profiles, with 74% of repeats located in the same cluster or having close (<5% of the embedding dimensions coordinates limits) geometric coordinates in the tSNE representation. There were 48 (26%) cases that exhibited less similar profiles among the independent NIPS repeats, which may indicate dynamic changes in cfDNA or transient status during pregnancies. To eliminate potential biased observation due to the inclusion of biological and technical replicates, analysis repeated with only the first sample from the series revealed a similar clustering pattern (Supplementary Fig. 2).

We hypothesized that the observed clusters might reflect different maternal and/or fetal pathologies or conditions. To explore this, we retrospectively reviewed the maternal clinical records for diagnoses of immune-mediated diseases. As presented in Fig. 1b, 17% (*n* = 25) of inconclusive cases owing to a high QS were found to have AID, including SLE, antiphospholipid syndrome (APS), hypothyroidism, primary Sjogren’s syndrome, Graves’ disease, RA, psoriasis, and spondyloarthritis. Among these 25, 13 women had SLE, of which 3 had APS, 1 had thrombosis, and 1 had psoriasis as a comorbidity. The estimated prevalence of SLE is 134.5 cases per 100,000 persons in the Caucasian population^30,31^. Based on this prevalence rate and the number of identified SLE cases, pregnant women with SLE had at least a sixty-fold higher chance of an inconclusive NIPS result than those without SLE (unadjusted odds ratio [OR] 63.4). In addition, 4% of cases (*n* = 5) had IBD. Given that the prevalence is 312 per 100,000 in women giving birth^32^, pregnant women with IBD have at least a 9-fold higher chance of an inconclusive NIPS result (unadjusted OR = 9.0) based on our cohort. Finally, 5% cases (*n* = 7) had blood disorders. In the low FF group, five (12%) inconclusive cases were associated with AID and two (5%) with blood disorders, respectively. Cluster annotation revealed a dominant representation of AID cases in cluster 4 (Fig. 1c). Investigation of only the first sample from inconclusive cases also demonstrated a high genome-wide similarity among a subset of AID (*n* = 10), particularly SLE (Supplementary Fig. 3). With that, 5 cases with unknown or healthy medical history, one case with IBD and another case with a blood disorder shared a common lineage with these AID cases (for simplicity, referred to as the AID clusters). Taken together, GIP*Xplore* analysis of the inconclusive cases identified a cluster of pregnancies enriched with AID (*P* < 1e-06). In the remaining clusters (13, 19 and 20), no common cause could be identified in the clinical records.

**Fig. 2:**
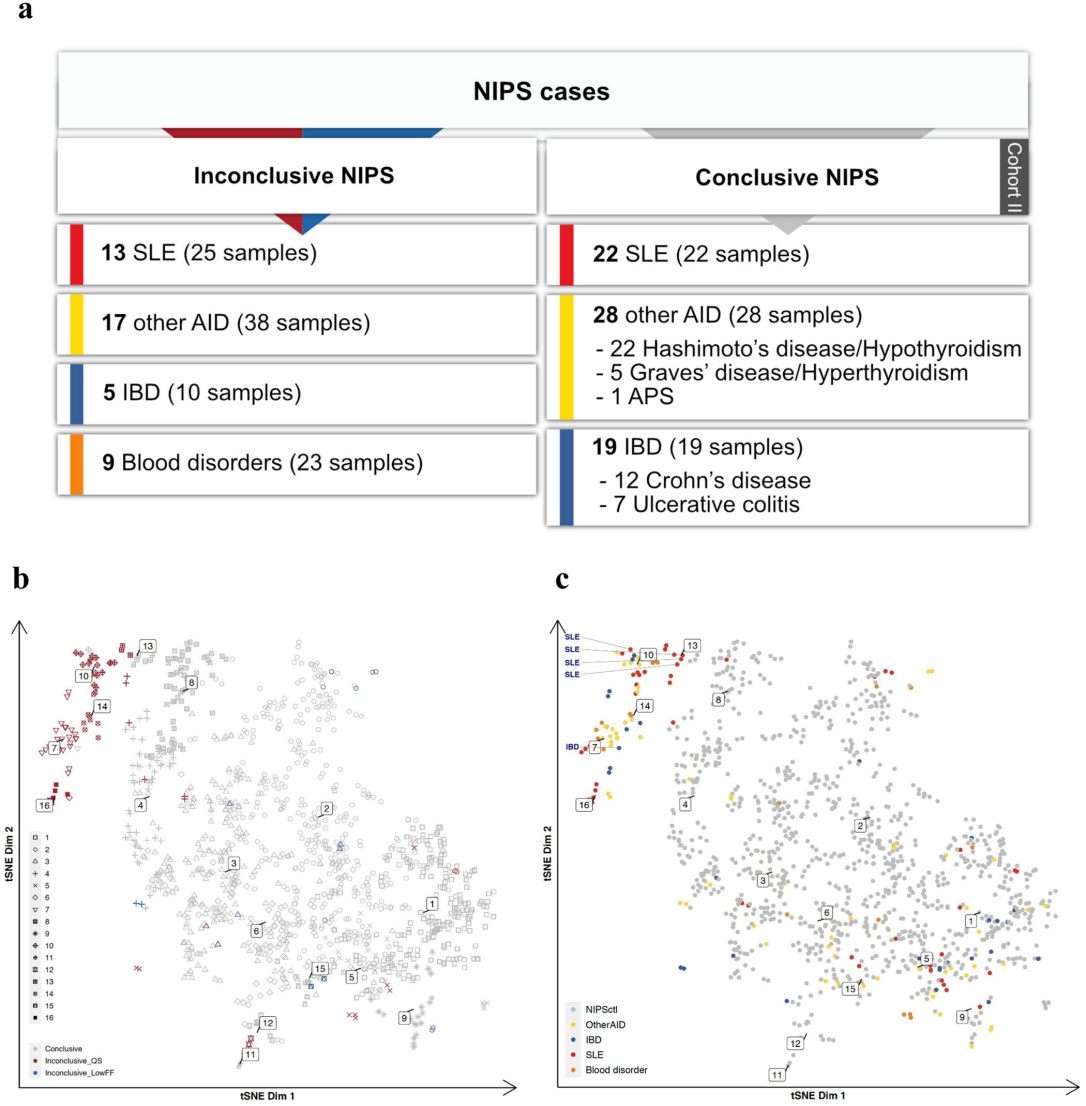
Analysis of AID and IBD from both inconclusive and conclusive NIPS. **a** summary of analysis cohort. **b** tSNE representation of the cohort, including AID and IBD NIPS with inconclusive and conclusive results. Point color indicates NIPS result. **c** the same tSNE representation as in **b**, with the annotation of phenotypes shown in different colors. NIPSctl represents NIPS samples with conclusive results. Five conclusive SLE and IBD NIPS cases that are colocalized with inconclusive AID and IBD samples are annotated additionally with text.

Next, we questioned whether the AID/IBD-specific signal could also be observed in pregnancies with a conclusive NIPS result. To explore this, we combined the cfDNA profiles of 44 inconclusive NIPS cases (96 samples) that were included in the aforementioned analysis from the Cohort I and additionally Cohort II - 69 conclusive NIPS cases from pregnant women with known diagnoses of AID (SLE, APS, hypothyroidism/Hashimoto’s disease, hyperthyroidism/Graves’ disease) or IBD (CD and UC; Fig. 2a). GIP*Xplore* on these cases together with control NIPS samples showed that disease-associated inconclusive NIPS profiles drifted away from the controls and formed sub-clusters as expected (Fig. 2b and Supplementary Fig. 4). More importantly, 4 SLE and 1 IBD cases that had a conclusive NIPS result co-localized with inconclusive cases in clusters 10, 13 and 7, respectively (Fig. 2c).

### GIP*Xplore* enables AID prediction using NIPS profiles

**Fig. 3:**
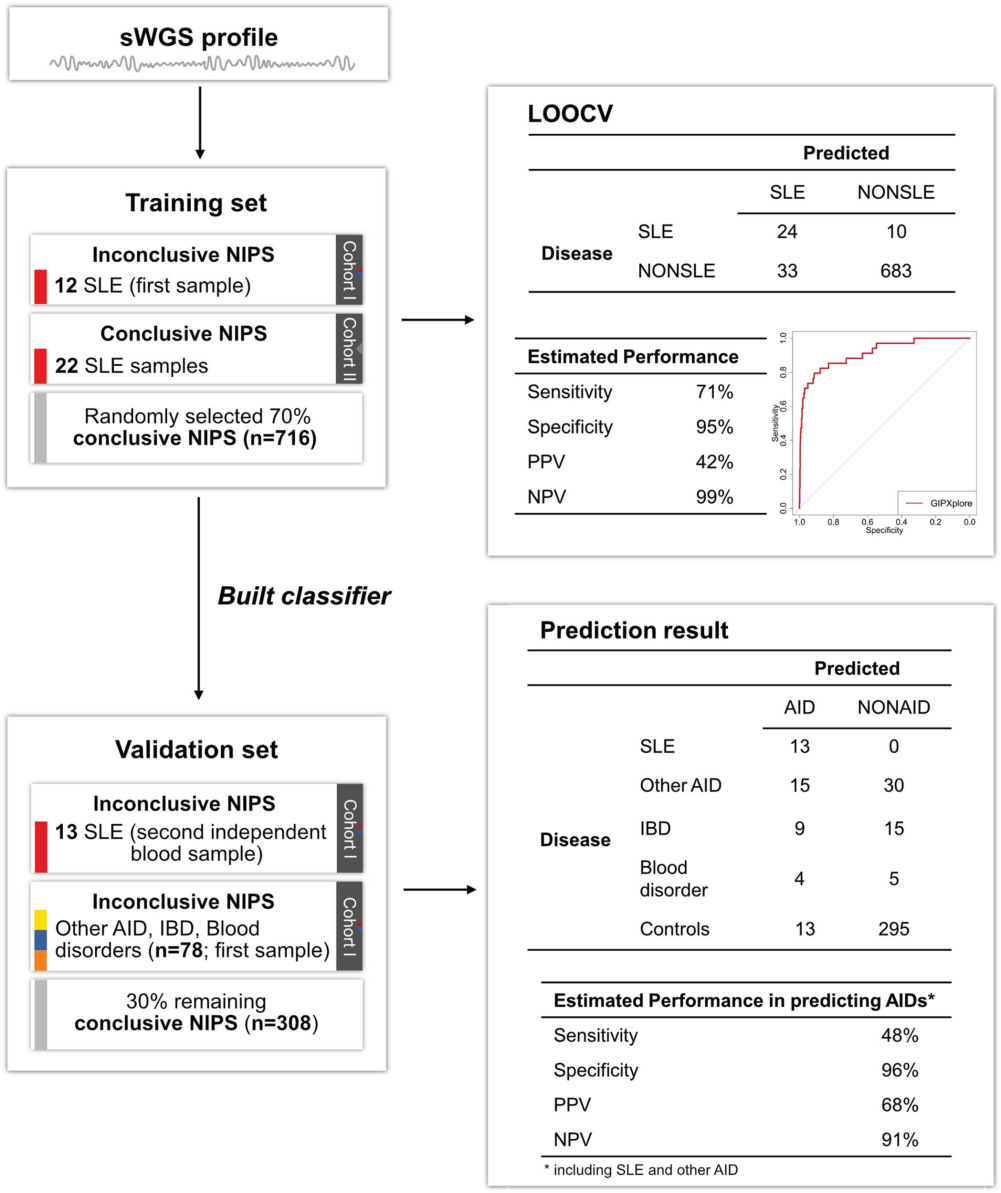
Workflow for building the classifier for AID prediction and the resulting performance. As one of the inconclusive SLE cases is the second pregnancy of the existing inconclusive case, we only included 12 inconclusive SLE cases in the training set. The one left out was used in the validation set as a ‘second’ SLE sample. In the prediction result for the validation set, performance characteristics (sensitivity, specificity, positive predictive value - PPV, negative predictive value - NPV) were calculated only using prediction results from AID (SLE and other AID) and controls.

Given that SLE is a prototypical autoimmune disease that is highly relevant to pregnancy management, we examined whether the genome-wide patterns observed in SLE could be used to identify pregnancies affected with SLE and/or pregnancies affected with other AID. We established a classifier trained on SLE cases and tested it using obstetric cfDNA data with and without disease conditions. We used a support vector machine (SVM) classifier based on genome-wide NIPS features. For this analysis, we grouped together SLE cases with both inconclusive (Cohort I) and conclusive (Cohort II) NIPS. SLE cases from Cohort I had more than one biological replicate. We split the NIPS cohort into the training set that included 12 inconclusive (Cohort I) and 22 conclusive (Cohort II) SLE cases, and the validation set (*n* = 399) that included the second biological repeat sample (Cohort I, *n* = 13) from each case in the training set (Fig. 3). First, we performed a predictive analysis in the training set to distinguish SLE from control NIPS profiles. Using leave-one-out cross-validation with pre-selected parameters, the estimated sensitivity was 71% at a specificity of 95%. The receiver operating characteristic (ROC) analysis showed an area under the curve (AUC) value of 0.91. A final classifier was then built on the training set. Applying the classifier on the independent validation set, all repeat SLE samples were correctly predicted. We also applied the final model to an independent set of cases with other AID, IBD and blood disorders. In concordance with the clustering analysis, where these cases could group with the SLE cases, 33%, 38% and 44% of profiles that primarily presented with other AID, IBD and blood disorders were predicted as SLE-like profiles. The specificities estimated in the training and validation set were consistent.

### Shared cfDNA profiles signatures in pregnant and non-pregnant populations with AID and IBD

We hypothesized that the disease-associated cfDNA genome-wide patterns observed in the obstetric population may also be characteristic to the general AID and IBD patient population. To test this, we investigated 24 SLE and 33 CD cfDNA samples from non-pregnant patients (Cohort III; Supplementary Data). The 24 non-pregnant SLE patients include 21 females and 3 males. Among 33 non-pregnant CD patients, 26 are males. We first performed the clustering analysis on these samples together with 104 cfDNA profiles from non-pregnant controls, in which two distinct disease-like clusters (4 and 7) formed, suggesting not only the presence of disease-associated cfDNA genome-wide patterns, but also potential stratification of SLE and CD profiles (Supplementary Fig. 5a, b). The separation of the clusters was not driven by sex (Supplementary Fig. 5c).

**Fig. 4:**
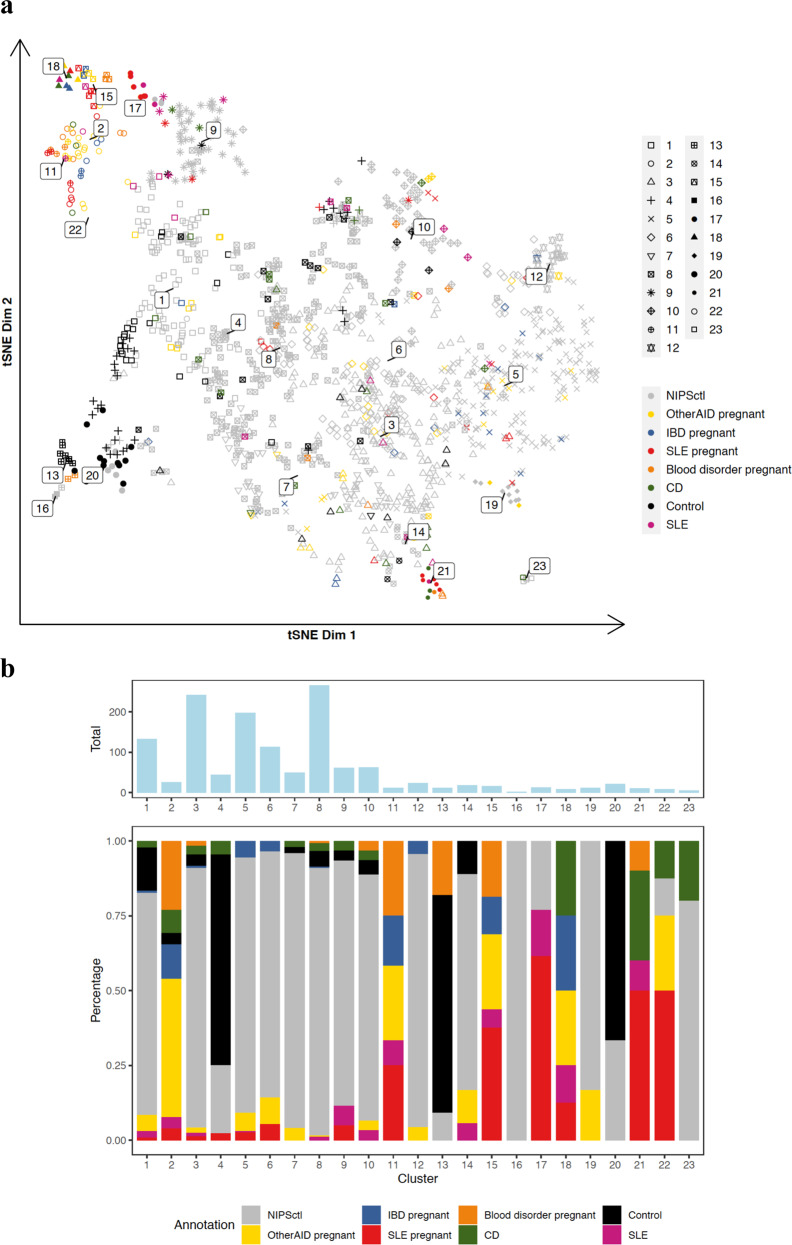
Clustering of cfDNA profiles from pregnant and non-pregnant AID and IBD. **a** tSNE representation of the analysis cohort. Point shape indicates the clusters that were defined by the Walktrap algorithm. Point color indicates phenotypic information. Control represents non-pregnant control samples. **b** upper bar plot shows the number of samples in each cluster and lower bar plot shows composition of samples in each cluster.

Next, using unsupervised clustering analysis, we assessed the similarity of disease-associated profiles in both non-pregnant and pregnant subjects. Interestingly, the unsupervised clustering revealed co-clustering of pregnant and non-pregnant subjects with AID and IBD (Fig. 4a, b). Seven non-pregnant SLE and eight non-pregnant CD cases were found in disease-associated clusters. Analogous to the SLE-associated clusters (Supplementary Fig. 4, clusters 13 and 16) identified in the previous analysis, both pregnant and non-pregnant SLE profiles assembled in clusters 11 and 17 (Fig. 4b). The shared cfDNA signatures in pregnant and non-pregnant patients presenting with AID and IBD were also evident in clusters 15, 18, 21 and 22. Moreover, one of the non-pregnant CD cases, clustered with AID in cluster 18, having AID (spondyloarthritis) comorbidity. Two other non-pregnant CD cases in cluster 3, grouping with AID, have other AID comorbidities (one with psoriasis and the other with autoimmune hemolytic anemia) as well. Other non-pregnant CD cases that clustered with AID were not associated with AID comorbidities. Cluster 21 was notably divergent from other AID and IBD clusters in multiple replicates of tSNE representations. Similar results were obtained in the cluster analysis excluding non-pregnant control subjects (Supplementary Fig. 6). We did not uncover any clinical factors that could have led to the separation using available information.

Though the classifier for AID prediction was not built for the general population, we reasoned that the disease-associated changes in the genome-wide profile of non-pregnant subjects could also be identified by the built classifier. When we applied the trained model on this non-pregnant cohort, a positive likelihood ratio (LR+) of 13 and negative likelihood ratio (LR-) of 0.52 for SLE, and a LR+ of 7.09 and LR- of 0.76 for CD were obtained (Supplementary Table 1). In the subsequent linear regression analysis, for non-pregnant SLE patients with high SLEDAI (Systemic Lupus Erythematosus Disease Activity Index), the odds of correct prediction increased by 17-fold (adjusted OR = 17.7), after adjusting for patient age, disease activity, and the existence of comorbidity. Disease activity is not significantly associated with the prediction outcome, after adjusting for the patient’s age, SLEDAI and the existence of comorbidity.

## Discussion

We demonstrate that the genome-wide cfDNA patterns of a subset of AID- and IBD-affected individuals can be distinguished from controls, and such cfDNA patterns can be used for the classification of immune-mediated diseases. The early detection of damage caused by SLE and other AID has been identified as key to improve long-term survival^33^. The potential to use cfDNA patterns as a non-invasive biomarker for immune-mediated diseases identification, paves the way towards screening, diagnosis and monitoring of AID and IBD. Since the patterns are similar in both pregnant and non-pregnant individuals and the disease-specific patterns can be identified during the first trimester of pregnancy, this opens the potential to predict the presence of AID and IBD following NIPS.

The observed similarity in cfDNA patterns across clinically distinct AID and IBD may reflect similarities in the inflammatory processes and/or cfDNA release and clearance mechanisms involved in these diseases. AID cause inflammation and damage to various tissues, often characterized by increased apoptosis and impaired clearance of apoptotic cells^11^. The impaired clearance of the cell death process can affect cfDNA levels and engage intracellular antigens that lead to auto-inflammatory responses^34^. The potential role of cfDNA in the pathogenesis of IBD has been previously suggested^10^. Multiple lines of evidence have suggested that cfDNA is mainly released via apoptosis in healthy individuals^35,36^. However, different types of programmed cell death, such as NETosis and pyroptosis, can also contribute to elevated cfDNA levels^9,11,37,38^. Particularly, NETosis, where neutrophils extrude DNA into the extracellular environment in the form of neutrophil extracellular traps (NETs)^39^, was found to be associated with the elevation of cfDNA levels in pathological conditions such as SLE and RA^40,41^. It has also been observed that NETs play an important role in IBD^42,43^, as well as other autoimmune and inflammatory comorbidities, including psoriasis, primary Sjogren’s syndrome and Hashimoto’s thyroiditis^11,44–46^. Interestingly, neutrophil NETs were found to be associated with coagulation and thus promote thrombosis^47^. In line with this, we demonstrated that cfDNA profiles with thrombosis appeared to colocalize with autoimmune diseases. Besides the release of cfDNA, the abnormal activity of DNase I and DNase I-like III (DNase IL3), two nucleases that carry out the digestion of nucleosomal DNA in plasma, have been associated with SLE and IBD^48–51^. Chan et al. (2014) have demonstrated that plasma cfDNA from SLE patients with an active disease has a distinct cfDNA profile characterized by fragment size shortening, as well as aberrant genomic and methylation patterns^48^. The level of abnormalities was correlated with anti-double-stranded DNA (anti-dsDNA) antibody levels, suggesting a potential association with the impaired clearance of cfDNA. However, the role of cfDNA in these complex diseases is yet to be fully explored, and the cause of shared qualitative cfDNA patterns in SLE and IBD patients observed in our study is not yet understood. Furthermore, SLE patients are more likely to have an altered genome-wide cfDNA profile with more heterogeneous subclusters than other autoimmune and inflammatory diseases. Though the numbers are small, interrogating non-pregnant SLE cases showed a putative association between cfDNA profile aberrations and a high Systemic Lupus Erythematosus Disease Activity Index (SLEDAI). Whether the aberration is the result of disease activity or pathogenic mechanisms requires further examination.

We demonstrate that AID and IBD during pregnancy are risk factors for an inconclusive NIPS. While AID and IBD may be associated with impaired cfDNA degradation process, a higher maternal cell turn-over resulting in low fetal fractions may also result in an inconclusive NIPS. Although other reports indeed showed AID to be a risk factor for inconclusive NIPS as a consequence of a reduced fetal fraction, we show that 83% of AID-related inconclusive NIPSs are actually due to atypical cfDNA sequencing profiles. While no study has investigated the association between inconclusive NIPS and IBD, we show that IBD may also exhibit altered cfDNA patterns. The potential to predict the presence of AID and IBD during the first trimester of pregnancy offers new opportunities for prenatal management following NIPS. Pregnancies with AID, particularly SLE, are considered high-risk for both mother and fetus. These risks include disease flares, pre-eclampsia, miscarriages, preterm birth, and intrauterine growth restriction^52,53^. New-onset SLE may develop during pregnancy, in which the detection can be complicated by pregnancy-related changes and the clinical manifestation might be more severe^54^. Women with known AID/IBD are referred to a high-risk obstetric unit with multidisciplinary care and an intensive pregnancy follow-up.

Physiological changes, such as pregnancy, can lead to a distinct sWGS cfDNA profile (Supplementary Fig. 7). In the context of AID and IBD, the pathological signals related to the presence of the disease appear to be dominant. That is, both pregnant and non-pregnant individuals with AID and IBD cluster together and are similarly classified, suggesting that the disease-associated cfDNA signatures override the pregnancy-related signatures. This is likely due to the fact that the majority of DNA fragments in maternal plasma are maternally derived. In both pregnant and non-pregnant cases, AID and IBD may show either altered or normal-like cfDNA profiles. It is unclear at this stage whether cases with altered profiles have worse outcomes in terms of pregnancy, treatment response, disease activity or other clinical parameters. Studies have investigated the impact of maternal medications (e.g. immunomodulatory therapy) on NIPS results, but there is no clear consensus. Some studies suggested that the use of immunomodulators may reduce FF and/or increase variance and thereby have an adverse effect on NIPS performance^55,56^. Other studies suggest that immunomodulators suppress disease activity and could therefore be expected to normalize NIPS profiles^24,27^.

Given the design of the current study, the clinical information for the inconclusive NIPS cohort is incomplete. We only partially unraveled the phenotypes linked to the clusters being identified. Future analysis of maternal conditions, and their impact on fetal health and pregnancy outcomes require a standardized model with systematic clinical data collection. This would allow further evaluation of the effects of AID and IBD on cfDNA patterning and its potential use in prenatal care. For early diagnosis in presymptomatic AID/IBD individuals at risk (e.g. first-degree relatives in multiplex IBD families)^57^, cfDNA genomic profiles might have a broader place as non-invasive biomarkers. Age- and gender-matched case and control cohort needs to be evaluated to eliminate potential bias. While we examined cfDNA samples collected from non-pregnant patients with different treatment and status of AID and IBD disease, collecting longitudinal cfDNA samples before and during the treatment course can shed more light on the association between the treatment and change of cfDNA profile. Large, prospective cohort studies are therefore warranted, which could explore its prognostic and predictive accuracy in immune-mediated disorders aiming to further individualize treatment strategies.

In conclusion, the ability to identify AID and IBD with cfDNA sequence patterns creates new opportunities for immune-mediated disease management. More importantly, using this approach during NIPS can identify pregnant women with higher risks of pregnancy complications and redirect prenatal management.

## Methods

### Study design and participants

The study retrospectively investigated NIPS data from a population of 81,611 pregnant women between July 2017 and December 2020. Peripheral blood was sampled in pregnant women from the 10th week of gestation onwards in Cell-Free DNA collection tubes (Roche Diagnostics). For the cases with an inconclusive result, a second blood sample was requested. Medical history of these repeated inconclusive cases was obtained by reviewing medical records from the referring clinical team with consent from 185 individuals. The study was approved by University Hospitals Leuven Ethics Committee (S62817).

Blood samples were collected from 24 and 33 non-pregnant patients with SLE and CD, respectively. Additional 104 non-pregnant subjects without known diagnosis of cancer (from self-reported questionnaires) and normal cfDNA profiles were included. Written consent was obtained from all participants in study S60268 and S62795, and both studies were approved by the University Hospitals Leuven Ethics Committee.

### Cell-free plasma DNA extraction and sequencing

Blood plasma was isolated through a standard two-step centrifugation procedure. cfDNA was extracted from 2 ml plasma using the Maxwell HT ccfDNA kit (Promega, Madison; automated procedure). DNA sequencing libraries of cfDNA were prepared using the KAPA HyperPrep kit (Roche Diagnostics). Whole-genome sequencing was carried out on a HiSeq2000, HiSeq2500, HiSeq4000, or NovaSeq sequencer (Illumina) generating 36 bp single-end or 51×2 paired-end reads. Raw reads were clipped to single-end 36 bp for standard processing and were mapped to the human reference genome GRCh38. Mean read count for downstream analysis was 9.96 million.

Sequencing data was subjected to the GIPSeq analysis pipeline as previously described^16^. In short, chromosomal z-scores are generated that measure the differential cfDNA representation, in standard deviations, between the sample and a reference set containing samples from women carrying a euploid fetus. A genome-wide quality score (QS), measuring the standard deviation of all autosomal z-scores following removal of the highest and lowest scoring chromosomes, was obtained. A NIPS result was marked as inconclusive when no reliable conclusion of fetal trisomy 21, 18 or 13 could be made, due to a low fetal fraction (<4%) or a deviating QS (QS ≥ 2) in the GIPSeq profile. Cases with inconclusive NIPS results at the second sample were included in the study. Repeated inconclusive NIPS results that were classified as being suggestive of an underlying malignancy or a maternal constitutional copy number variation were excluded from the analysis. Among the selected inconclusive case series, two cases (one cfDNA sample from a pregnant woman diagnosed with SLE and one diagnosed with immune thrombocytopenic purpura) only had the first sample available. These two pregnant women both had repeated NIPS results in a previous pregnancy and refused a second sampling after the first inconclusive NIPS in the following pregnancy.

Smoothed bin counts (57,509 autosome bin features) from GIPSeq were further used for GIP*Xplore* analysis. Principal component analysis (PCA) was used for dimension reduction and the top 50 principal components (PCs) were extracted for distance matrix construction using the Euclidean distance, followed by Walktrap community detection to define clusters with fixed parameters (the nearest number of nodes was 8 with a walk step of 2 and the subsequent optimal number of communities was determined by modularity). To visualize the dataset in lower dimensions, t-distributed stochastic neighbor embedding (tSNE) was used. For hierarchical clustering, the top 50 PCs and the Euclidean distances were used, and the hierarchical tree was constructed using Ward’s linkage. PCA transformed genome-wide features were used for training in the machine learning model. Given that PCA was performed on the training data, the test data was projected onto the training PCA space for classification tasks. Performance was estimated by leave-one-out cross-validation and receiver operating characteristic (ROC) analysis. For the classifier, we used a support vector machine (SVM) and hyperparameters were chosen based on the grid search with 90% of the training data. Weighted sample size was accounted for in the model for imbalanced classes.

### Statistical analysis

The proportion of conclusive and inconclusive (owing to a high QS) results were compared between pregnant women with and without AID using the odds ratio. The number of expected AID cases in the NIPS cohort was estimated using the population prevalence. Analogously, the odds ratio for pregnancy-induced IBD was computed. Outcomes of a high QS or a low FF among pregnant women with obesity and without obesity were compared using odds ratio as well. Logistic regression analysis was performed to determine an association between a given cluster and the proportion of AID cases. A *P* value was reported for clusters that were significantly enriched for cases with AID. A logit model was used to determine the log odds of the SLE prediction result in non-pregnant SLE cases with patient age, SLEDAI (Systemic Lupus Erythematosus Disease Activity Index) and disease activity as the independent variables. SLEDAI was categorized into four levels: not available, no activity (0), mild (1–5), and high (>6). Disease activity was categorized into two levels: yes (low, moderate, and high) and no.

### Reporting summary

Further information on research design is available in the Nature Research Reporting Summary linked to this article.

## Supplementary information

Supplementary Material

Supplemental dataset

Reporting Summary

## Data Availability

Processed alignments of sequencing data are archived to ArrayExpress (https://www.ebi.ac.uk/arrayexpress/) with unrestricted access under accession number E-MTAB-11607 and E-MTAB-10934. All other materials associated with this study are present in the paper or the Supplementary Information.

## Code availability

Code can be available upon reasonable request to the corresponding author.
