# Supplementary material for: Machine learning-based detection of immune-mediated diseases from genome-wide cell-free DNA sequencing datasets
Source: NPJ Genom Med. 2022 Sep 14;7:55. doi: 10.1038/s41525-022-00325-w (PMC9470560; doi:10.1038/s41525-022-00325-w)
Supplement: Supplementary file 1 — Supplementary Material [file 41525_2022_325_MOESM1_ESM.docx]

**Supplementary Figure 1. Clustering of inconclusive NIPS cohort.**

**
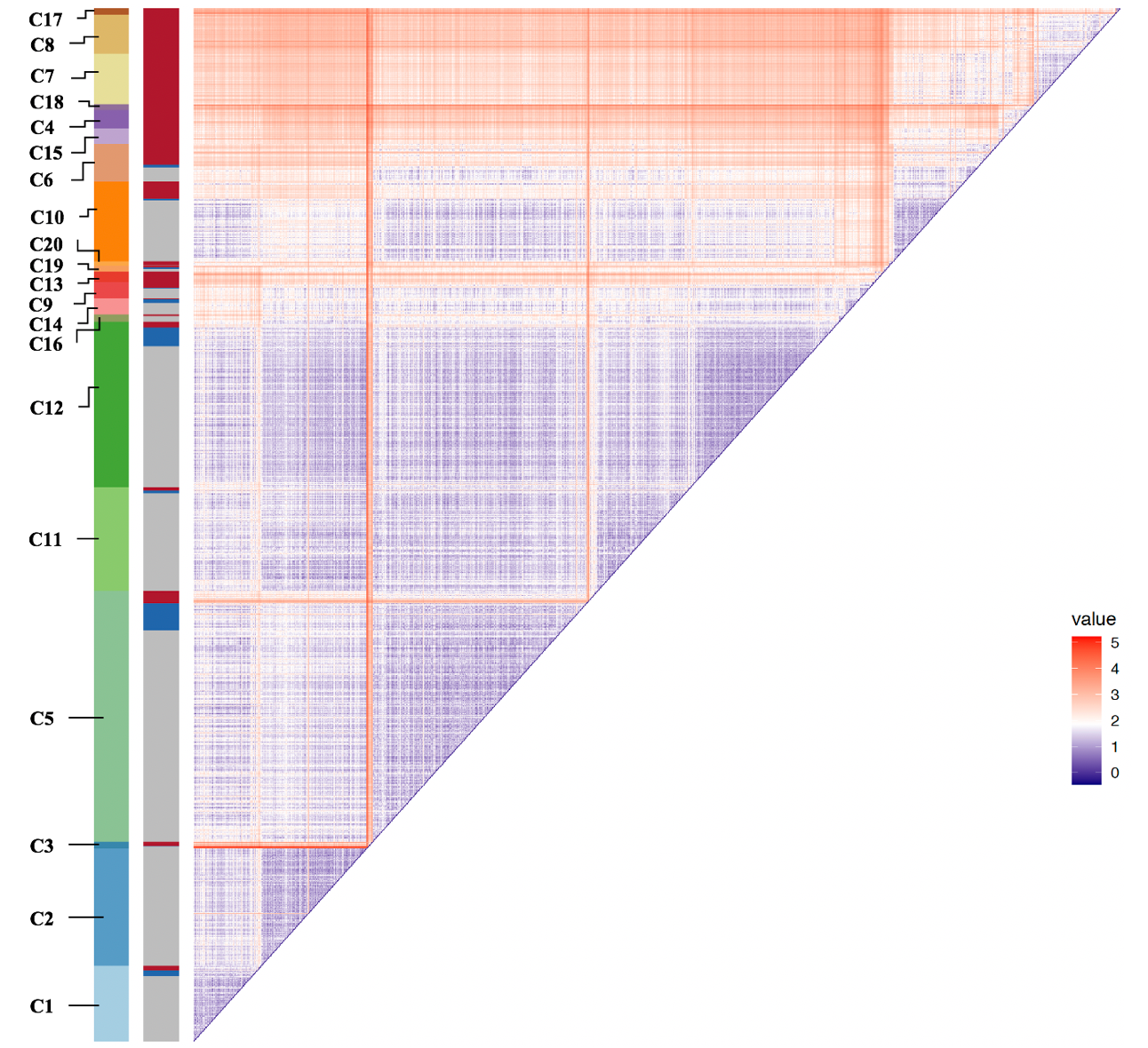
**

**Supplementary Figure 1.** The distance matrix was sorted by clusters defined by the Walktrap as shown in Figure 1a. In the distance matrix, the higher similarity between two profiles, the lower the value (purple). The left bar colors indicate the cluster that a sample belongs to. The second left bar indicates NIPS result for a sample, with conclusive NIPS in gray, inconclusive NIPS owing to a low fetal fraction in blue and inconclusive NIPS owing to a high genome-wide quality score (QS) in red.

**Supplementary Figure 2. Clustering with only the first sample of the repeated inconclusive cases.**

**
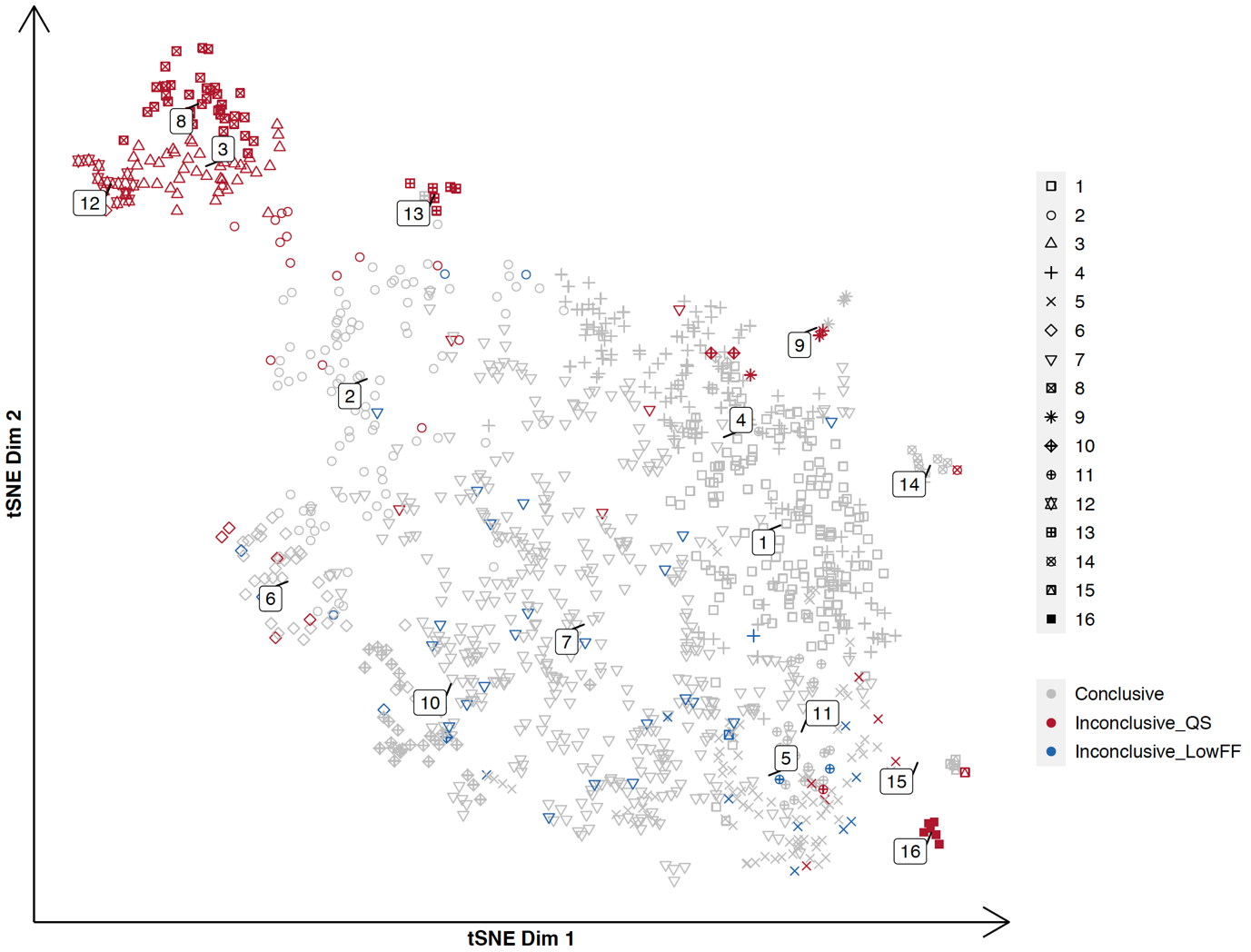
**

**Supplementary Figure 2.** tSNE representation of the first inconclusive samples (n=185) and conclusive NIPS (n=1024). Each point represents one sample. Color red and blue indicate inconclusive samples due to deviating QS and low FF, respectively. Point shape represents the clusters being identified using Walktrap community detection.

**Supplementary Figure 3. AID clusters.**

**
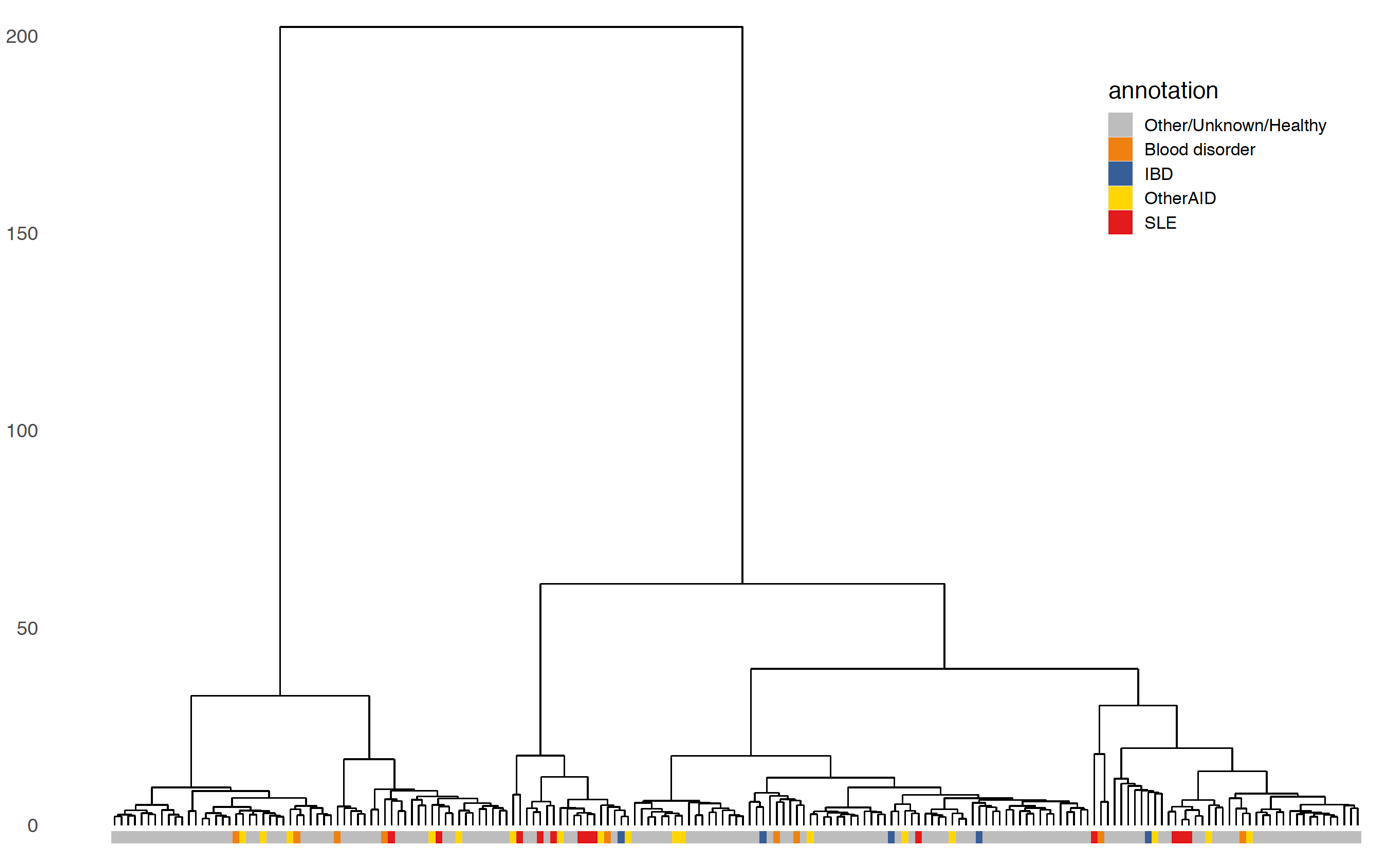
**

**Supplementary Figure 3.** Hierarchical clustering of the first sample of inconclusive cases (n=185).

**Supplementary Figure 4. Clustering of the AID and IBD from both conclusive and inconclusive NIPS.**

**
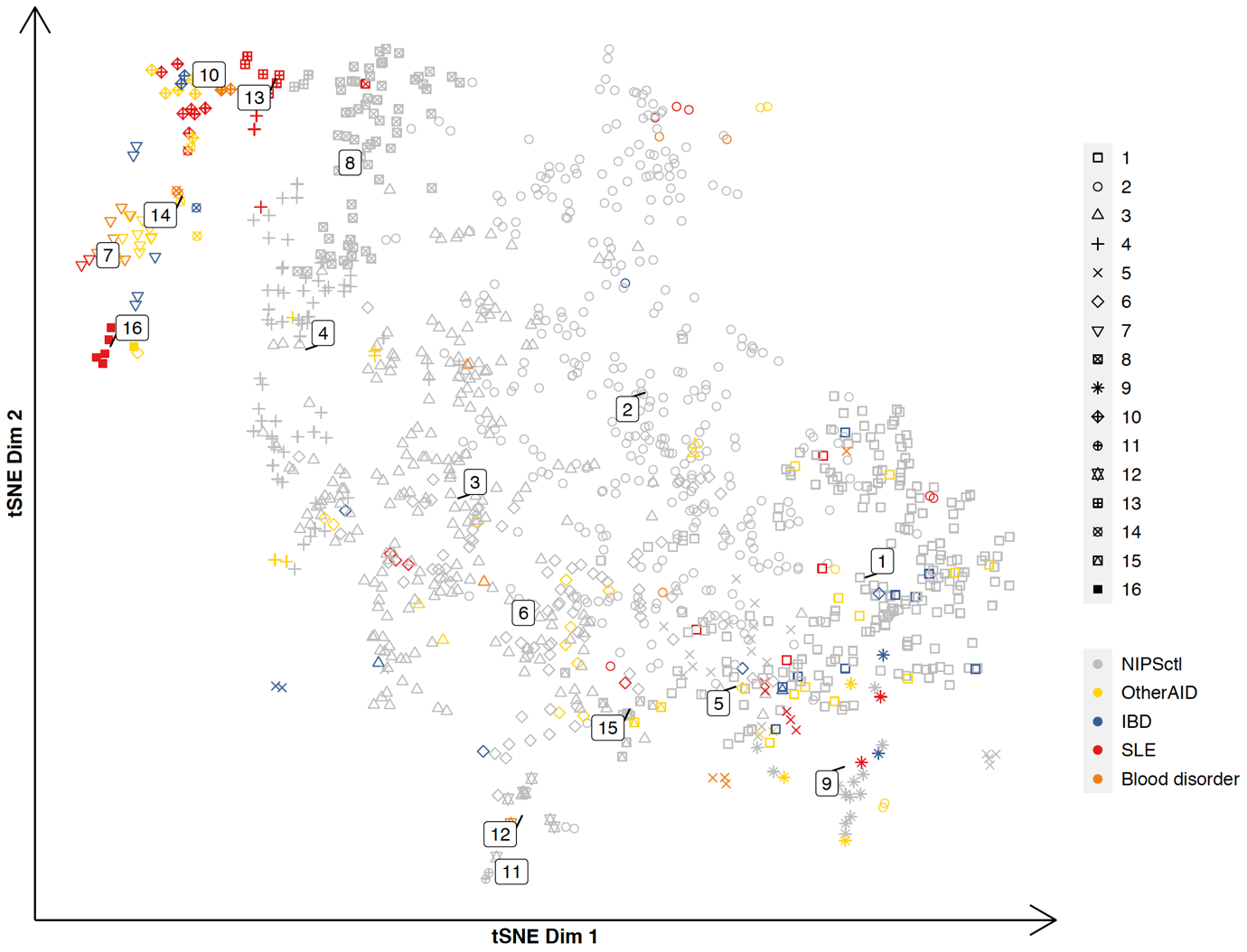
**

**Supplementary Figure 4.** Clusters of each sample are noted with shape of the point.

**Supplementary Figure 5. Clustering on non-pregnant SLE and IBD.**

**a**

**
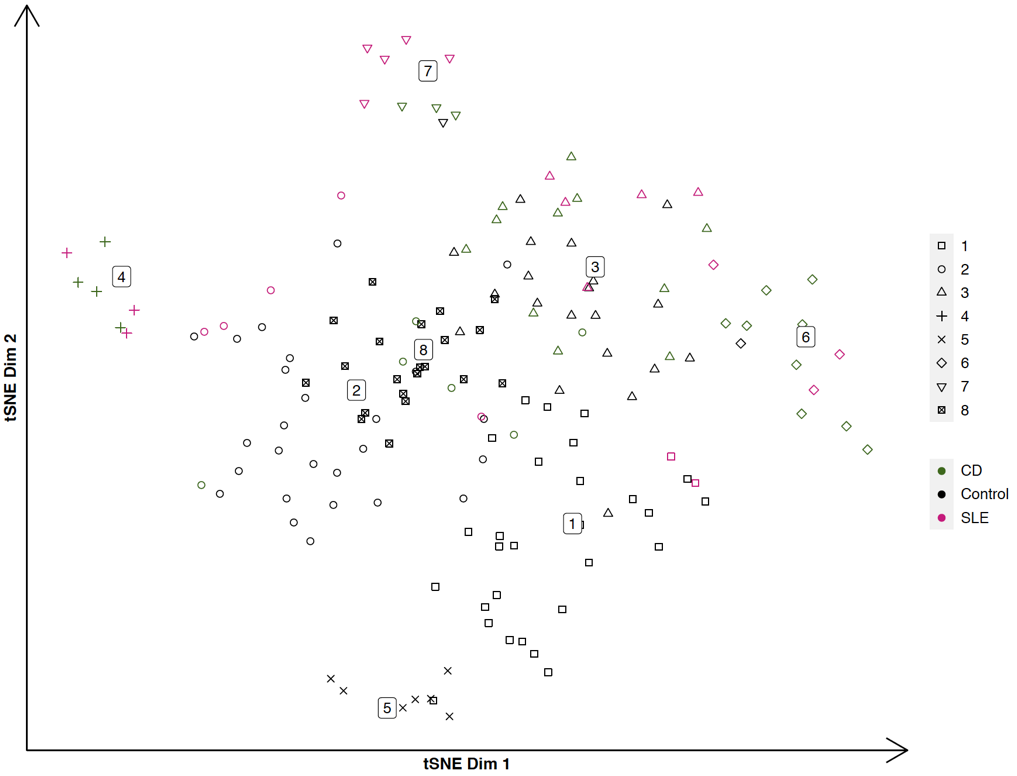
**

**b**

**
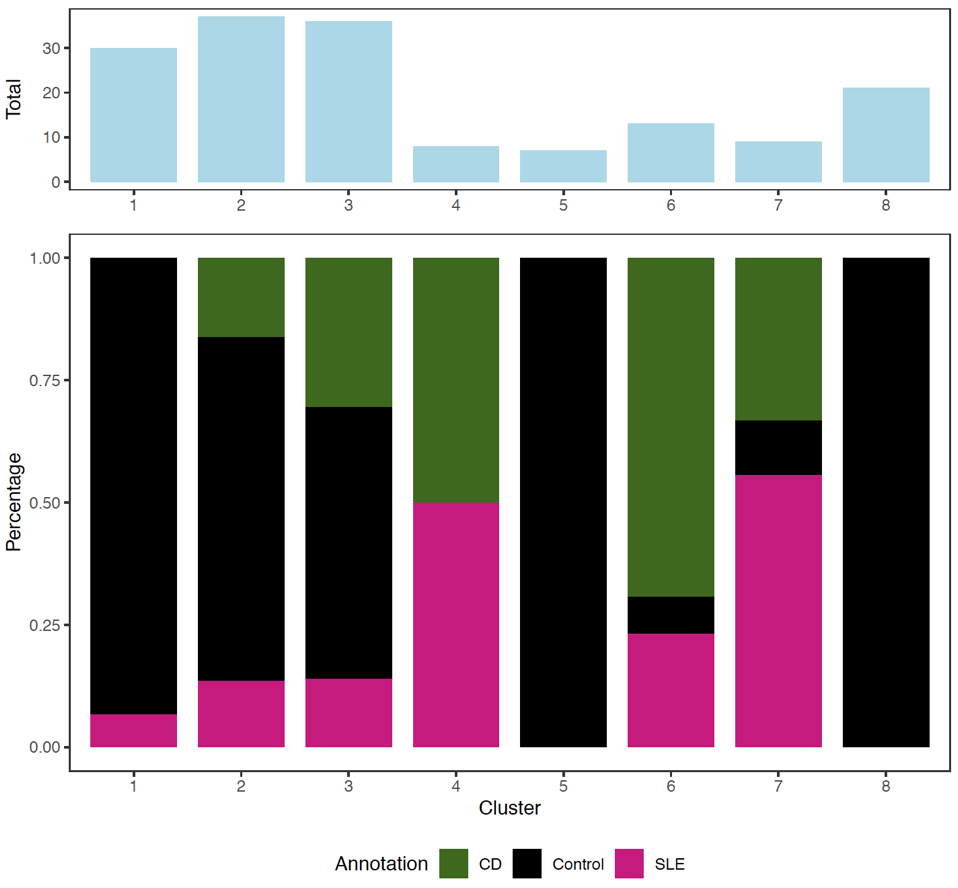
**

**c**

**
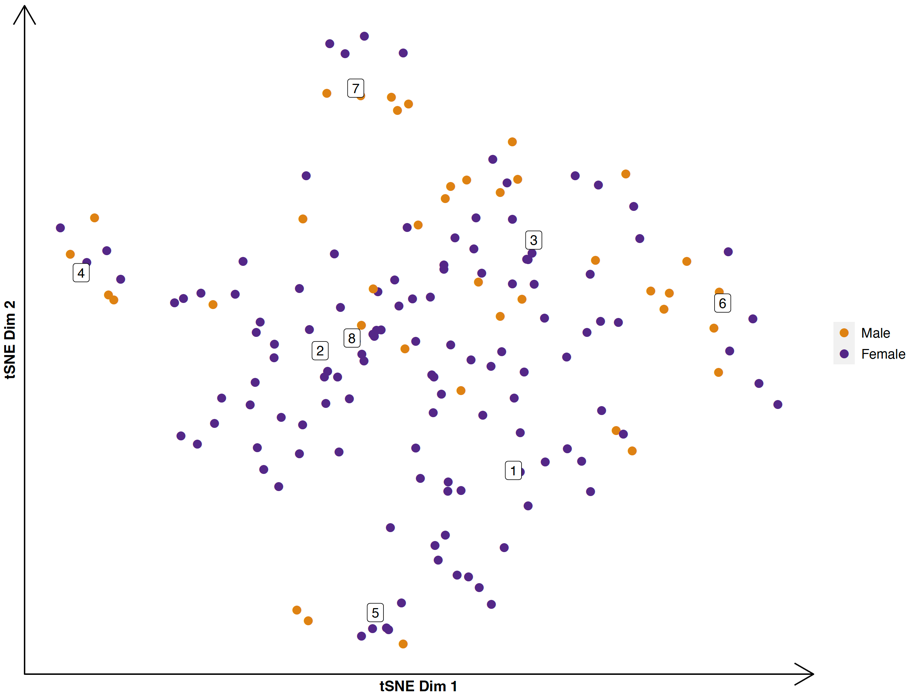
**

**Supplementary Figure 5.** The analysis was performed only using non-pregnant subjects, including 24 SLE, 33 IBDs and 104 non-pregnant control cfDNA samples. **a**, tSNE representation. **b**, upper bar plot shows the number of samples in each cluster and lower bar plot shows composition of samples in each cluster. **c**, tSNE representation from panel **a** with sex annotation.

**Supplementary Figure 6. Clustering of pregnant and non-pregnant SLE and IBD.**

**a**

**
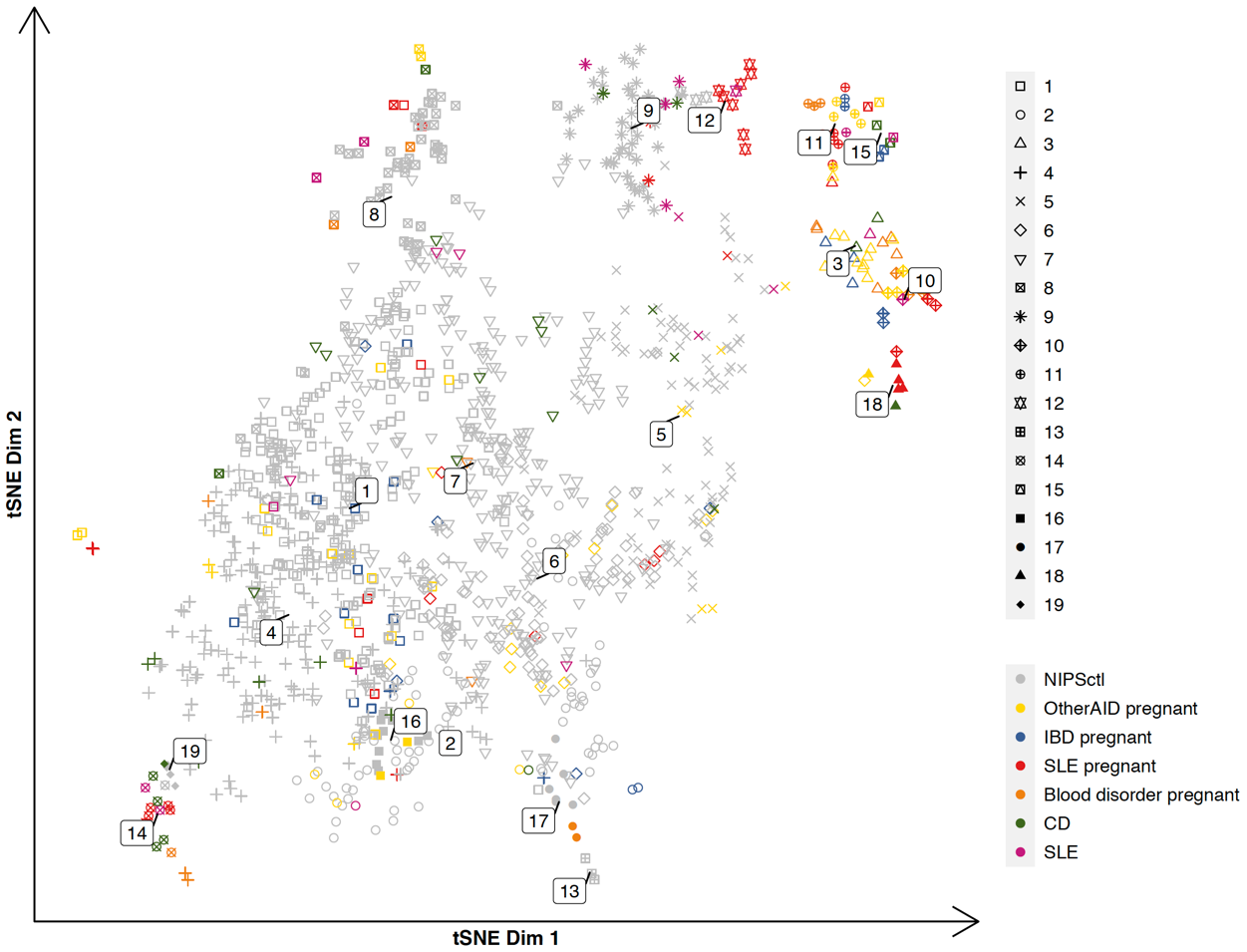
**

**b**

**
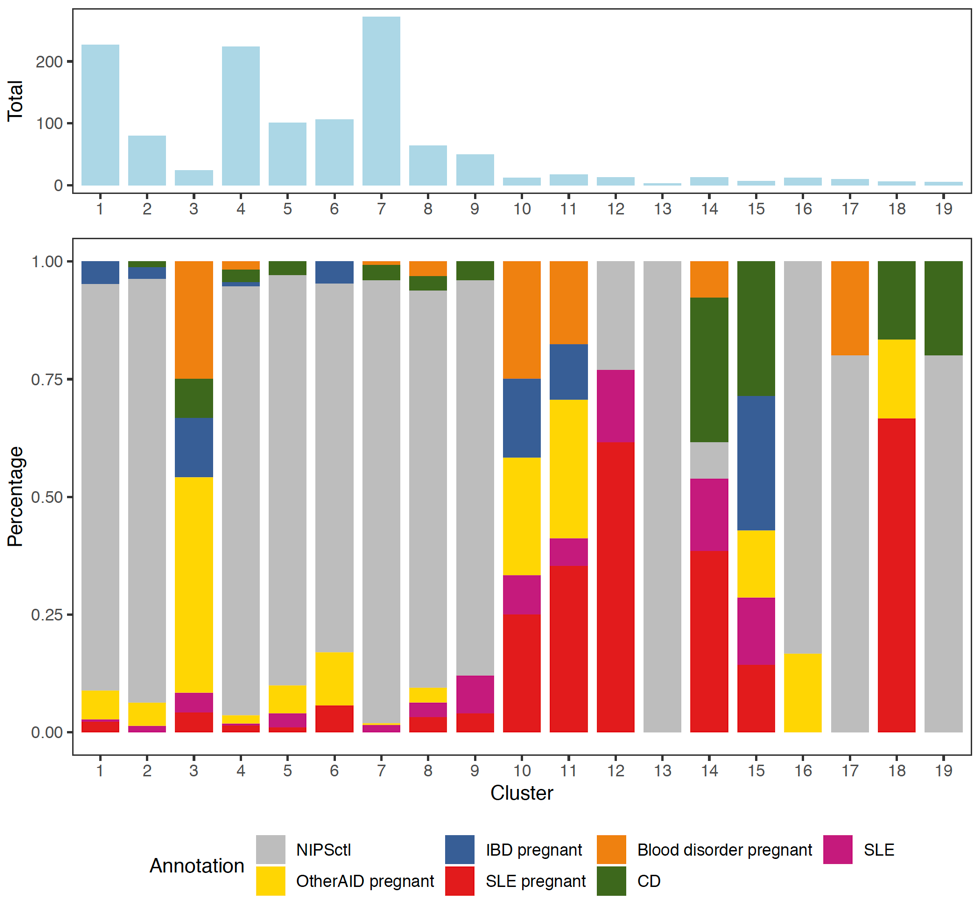
**

**Supplementary Figure 6.** The analysis on cfDNA profiles from both pregnant and non-pregnant AID/IBD cases with pregnant cases (NIPSctl) used as a control.

**Supplementary Figure 7. Pregnancy-induced changes in plasma cfDNA are reflected in genome-wide cfDNA profiles.**

**a**

**
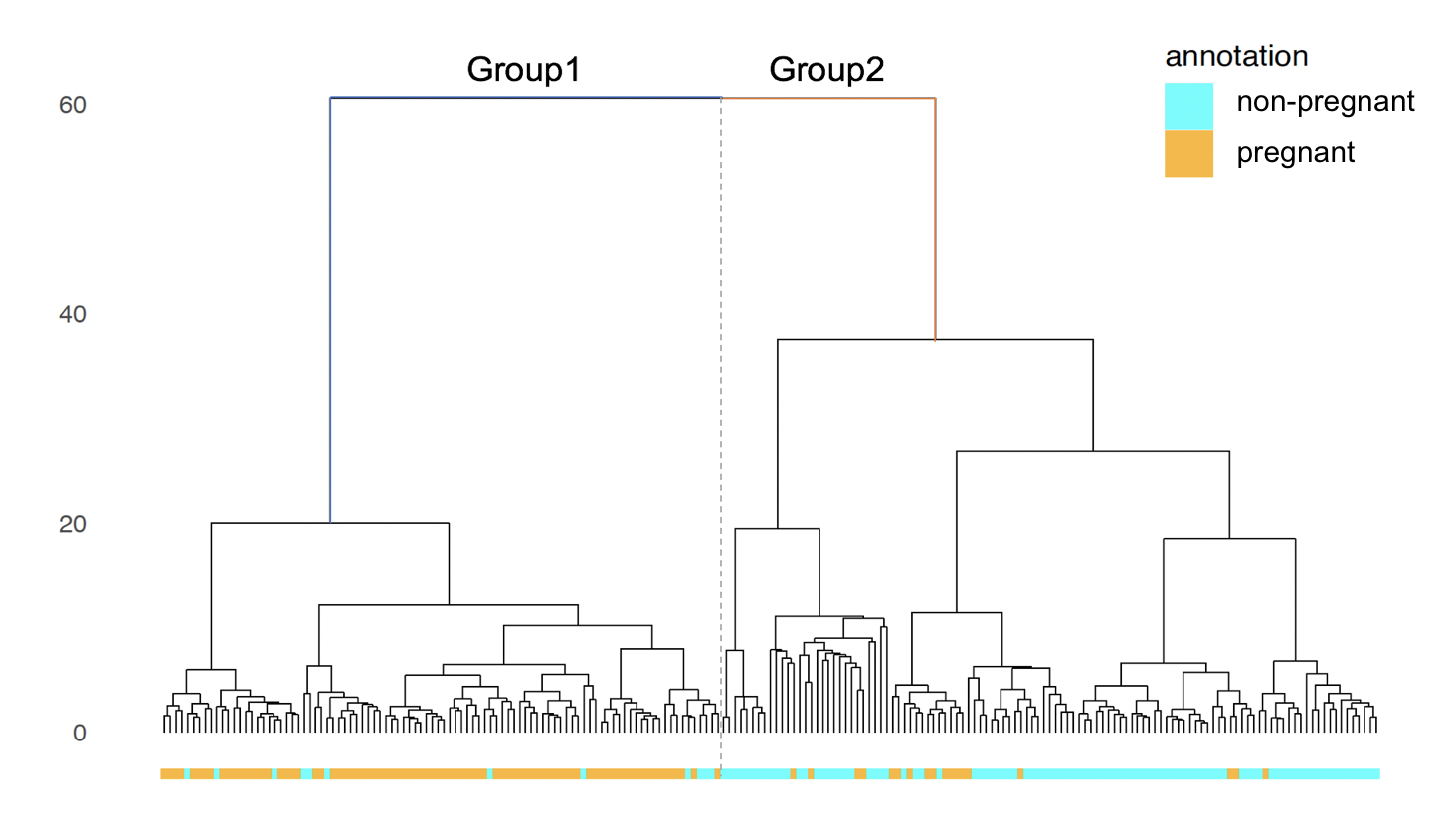
**

**b**

**
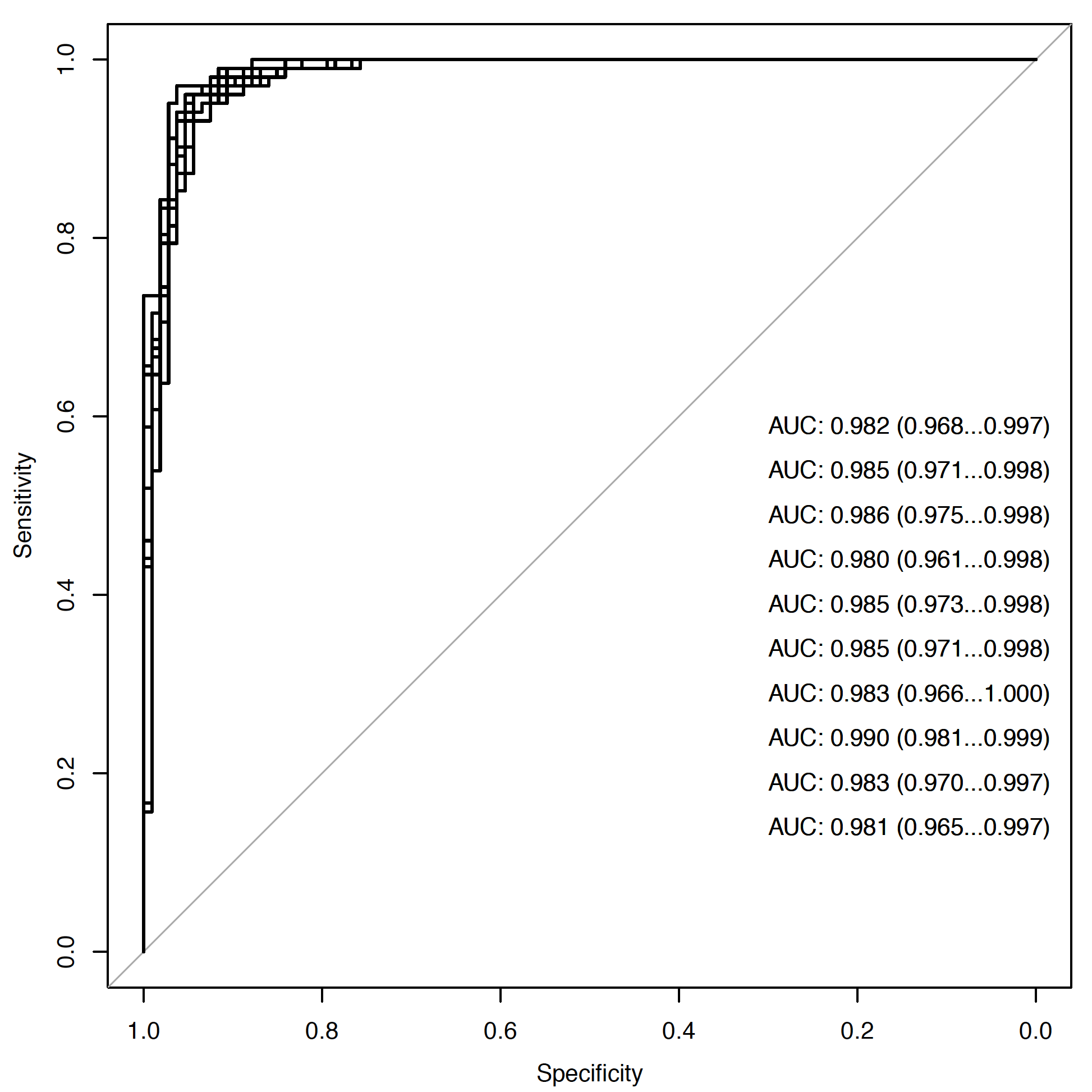
**

**Supplementary Figure 7. a**, agglomerative hierarchical clustering showed two groups enriched with pregnant (yellow) and non-pregnant healthy (cyan) profiles. Group 1 branch indicated pregnant profiles associated cluster, and Group 2 indicated non-pregnant healthy profiles associated cluster. **b**, ROC curves for discrimination between pregnant and non-pregnant profiles. Ten iterations of cross validation results were presented. AUC value and its confidence interval of each iteration were denoted.

**Supplementary Table 1**

|  |  | **Predicted** | |
| --- | --- | --- | --- |
|  |  | **AID** | **NONAID** |
| **Disease** | **SLE non-pregnant** | **12** | **12** |
|  | **CD non-pregnant** | **9** | **24** |
|  | **Control non-pregnant** | **4** | **100** |
