# Supplementary material for: Machine learning-based detection of immune-mediated diseases from genome-wide cell-free DNA sequencing datasets
Source: NPJ Genom Med. 2022 Sep 14;7:55. doi: 10.1038/s41525-022-00325-w (PMC9470560; doi:10.1038/s41525-022-00325-w)
Supplement: Supplementary file 3 — Reporting Summary [file 41525_2022_325_MOESM3_ESM.pdf]

nature portfolio

Corresponding author(s): Joris Robert Vermeesch

Last updated by author(s): Mar 28, 2022

Reporting Summary

Nature Portfolio wishes to improve the reproducibility of the work that we publish. This form provides structure for consistency and transparency in reporting. For further information on Nature Portfolio policies, see our Editorial Policies and the Editorial Policy Checklist.

Statistics

For all statistical analyses, confirm that the following items are present in the figure legend, table legend, main text, or Methods section.

n/a	Confirmed	
☐	☒	The exact sample size (n) for each experimental group/condition, given as a discrete number and unit of measurement
☐	☒	A statement on whether measurements were taken from distinct samples or whether the same sample was measured repeatedly
☐	☒	The statistical test(s) used AND whether they are one- or two-sided<br>*Only common tests should be described solely by name; describe more complex techniques in the Methods section.*
☐	☒	A description of all covariates tested
☐	☒	A description of any assumptions or corrections, such as tests of normality and adjustment for multiple comparisons
☐	☒	A full description of the statistical parameters including central tendency (e.g. means) or other basic estimates (e.g. regression coefficient) AND variation (e.g. standard deviation) or associated estimates of uncertainty (e.g. confidence intervals)
☐	☒	For null hypothesis testing, the test statistic (e.g. F, t, r) with confidence intervals, effect sizes, degrees of freedom and P value noted<br>*Give P values as exact values whenever suitable.*
☒	☐	For Bayesian analysis, information on the choice of priors and Markov chain Monte Carlo settings
☒	☐	For hierarchical and complex designs, identification of the appropriate level for tests and full reporting of outcomes
☒	☐	Estimates of effect sizes (e.g. Cohen's d, Pearson's r), indicating how they were calculated

Our web collection on statistics for biologists contains articles on many of the points above.

Software and code

Policy information about availability of computer code

Data collection	R software 3.6
Data analysis	Raw reads were clipped to single-end 36 bp for standard processing and were mapped to the human reference genome GRCh38. Smoothed bin counts (57,509 autosome bin features) were used for analysis. Principal component analysis (PCA) was used for dimension reduction and the top 50 principal components (PCs) were extracted for distance matrix construction using the Euclidean distance, followed by Walktrap community detection to define clusters with fixed parameters (the nearest number of nodes was 8 with a walk step of 2 and the subsequent optimal number of communities was determined by modularity). To visualize the dataset in lower dimensions, t-distributed stochastic neighbor embedding (tSNE) was used. For hierarchical clustering, the top 50 PCs and the Euclidean distances were used, and the hierarchical tree was constructed using Ward's linkage. PCA transformed genome-wide features were used for training in the machine learning model. Given that PCA was performed on the training data, the test data was projected onto the training PCA space for classification tasks. Performance was estimated by leave-one-out cross validation (LOOCV) and receiver operating characteristic (ROC) analysis. For the classifier, we used a support vector machine (SVM) and hyperparameters were chosen based on the grid search with 90% of the training data. Weighted sample size was accounted for in the model for imbalanced classes.

For manuscripts utilizing custom algorithms or software that are central to the research but not yet described in published literature, software must be made available to editors and reviewers. We strongly encourage code deposition in a community repository (e.g. GitHub). See the Nature Portfolio guidelines for submitting code & software for further information.

Data

Policy information about availability of data

All manuscripts must include a data availability statement. This statement should provide the following information, where applicable:

- Accession codes, unique identifiers, or web links for publicly available datasets
- A description of any restrictions on data availability
- For clinical datasets or third party data, please ensure that the statement adheres to our policy

Processed alignments of sequencing data are archived to ArrayExpress (https://www.ebi.ac.uk/arrayexpress/) with unrestricted access under accession number E-MTAB-11607 and E-MTAB-10934.

Field-specific reporting

Please select the one below that is the best fit for your research. If you are not sure, read the appropriate sections before making your selection.

☒ Life sciences ☐ Behavioural & social sciences ☐ Ecological, evolutionary & environmental sciences

For a reference copy of the document with all sections, see nature.com/documents/nr-reporting-summary-flat.pdf

Life sciences study design

All studies must disclose on these points even when the disclosure is negative.

Sample size	For pregnant patients, 185 inconclusive NIPS cases were included after data filtering and patient consent. Two to three conclusive control samples were matched to the sample processing time, sequencing batch and library preparation kit of inconclusive cases, accumulating to 1024 samples. For non-pregnant patients, available samples were included.
Data exclusions	Patients declined to participate into the study.
Replication	NA.
Randomization	NA.
Blinding	NA.

Reporting for specific materials, systems and methods

We require information from authors about some types of materials, experimental systems and methods used in many studies. Here, indicate whether each material, system or method listed is relevant to your study. If you are not sure if a list item applies to your research, read the appropriate section before selecting a response.

Materials & experimental systems

n/a	Involved in the study	
☒	☐	Antibodies
☒	☐	Eukaryotic cell lines
☒	☐	Palaeontology and archaeology
☒	☐	Animals and other organisms
☐	☒	Human research participants
☒	☐	Clinical data
☒	☐	Dual use research of concern

Methods

n/a	Involved in the study	
☒	☐	ChIP-seq
☒	☐	Flow cytometry
☒	☐	MRI-based neuroimaging

Human research participants

Policy information about studies involving human research participants

Population characteristics	Pregnant women with reproductive age were included in the study. Detailed age, ethnicity and phenotypic information were provided in the manuscript materials.
Recruitment	The study retrospectively investigated NIPS data from a population of 81,611 pregnant women between July 2017 and December 2020. Medical history of these repeated inconclusive cases was obtained by reviewing medical records from the referring clinical team with consent from 185 individuals. The study was approved by University Hospitals Leuven Ethics Committee (S62817).

Blood samples were collected from 24 and 33 non-pregnant patients with SLE and CD, respectively. Additional 104 non-pregnant subjects without known diagnosis of cancer (from self-reported questionnaires) and normal cfDNA profiles were included. Written consent was obtained from all participants in study S60268 and S62795, and both studies were approved by the University Hospitals Leuven Ethics Committee.

Ethics oversight	University Hospitals Leuven Ethics Committee

Note that full information on the approval of the study protocol must also be provided in the manuscript.
